# Impact of the McDonald Criteria 2017 on Early Diagnosis of Relapsing-Remitting Multiple Sclerosis

**DOI:** 10.3389/fneur.2019.00188

**Published:** 2019-03-15

**Authors:** Philipp Schwenkenbecher, Ulrich Wurster, Franz Felix Konen, Stefan Gingele, Kurt-Wolfram Sühs, Mike P. Wattjes, Martin Stangel, Thomas Skripuletz

**Affiliations:** ^1^Clinical Neuroimmunology and Neurochemistry, Department of Neurology, Hannover Medical School, Hannover, Germany; ^2^Department of Diagnostic and Interventional Neuroradiology, Hannover Medical School, Hannover, Germany

**Keywords:** multiple sclerosis, clinically isolated syndrome, McDonald criteria, MRI, oligoclonal bands, cerebrospinal fluid

## Abstract

Multiple sclerosis is a chronic immune mediated demyelinating disease leading to neurological disabilities that need to be diagnosed and treated early. Guidelines on multiple sclerosis diagnosis and monitoring experienced comprehensive changes over the last decades. The first McDonald criteria published in 2001 emphasized the importance of MR imaging but also recognized the role of cerebrospinal fluid diagnostics. The demonstration of an intrathecal immunoglobulin G synthesis is a well-established additional component and has a long tradition in the diagnosis of relapsing-remitting multiple sclerosis. However, the role of cerebrospinal fluid for diagnostic purposes was rather diminished in each revision of the McDonald criteria. In the latest revision of the McDonald criteria of 2017, the detection of an intrathecal immunoglobulin G synthesis as oligoclonal bands experienced a revival. Patients with the first clinical event suggesting multiple sclerosis who fulfill the criteria for dissemination in space can be diagnosed with relapsing-remitting multiple sclerosis when oligoclonal bands in cerebrospinal fluid are detected. The diagnostic sensitivity of these novel criteria with a focus on dissemination in time and oligoclonal bands as a substitute for dissemination in time was published in different cohorts in the last year and is of special interest in this review. Recently published data show that by applying the 2017 McDonald criteria, multiple sclerosis can be diagnosed more frequently at the time of first clinical event as compared to the 2010 McDonald criteria. The main effect was due to the implementation of oligoclonal bands as a substitute for dissemination in time. However, careful differential diagnosis is essential in patients with atypical clinical manifestations to avoid misdiagnoses.

## Introduction

Multiple sclerosis is the most frequent chronic inflammatory demyelinating disease in young adult leading to long term disability (1). Multiple sclerosis is characterized by inflammation in different regions of the central nervous system which is called dissemination in space (DIS) (2–6). Furthermore, inflammation of the central nervous system has to be recurring which is called dissemination in time (DIT) (2–6). Both criteria DIS and DIT have to be fulfilled either by clinical disease course with relapses and different neurological symptoms or by magnetic resonance imaging (MRI) demonstrating inflammatory lesions in different regions and different activity stages to diagnose multiple sclerosis (3–6). Since disease-modifying therapies administered in an early stage of multiple sclerosis have the potential to prevent relapses and future disabilities, an early diagnosis is essential (7–10). The McDonald diagnostic criteria for relapsing-remitting multiple sclerosis facilitated an early and accurate diagnosis in clinical practice (4, 6, 10, 11). In 85% of multiple sclerosis patients, the clinical manifestations start with a clinically isolated syndrome, the first clinical episode of the chronic inflammatory demyelinating disease (10, 12). Multiple sclerosis can be diagnosed when a typical clinically isolated syndrome is followed by a new clinical event with new symptoms which would be then considered as the second relapse. Alternatively, one or more new T2 and/or contrast enhancing lesions on a follow-up MRI scan could also demonstrate DIT allowing the diagnosis of multiple sclerosis in these patients when lesions in different regions of the central nervous system have also be found in one of the MRI scans or when the patient experienced symptoms related to different regions (4–6, 10). Since the introduction of the McDonald criteria of 2010 multiple sclerosis can be diagnosed based on a single baseline MRI scan showing at least one asymptomatic contrast enhancing lesion and non-enhancing lesions (5). The revised McDonald criteria of 2017 contain several novelties in the diagnosis of multiple sclerosis (2, 13). The criteria are easier to apply than the 2010 McDonald criteria, since it is no longer necessary to differentiate between cortical and juxtacortical MRI lesions and between symptomatic and asymptomatic contrast enhanced MRI lesions to fulfill the criterion for DIS (2). Further changes are that cortical lesions and symptomatic brainstem and spinal lesions can be used to demonstrate DIS (2). DIT can be demonstrated by contrast enhanced lesions independently whether they are asymptomatic or symptomatic, which has been shown to increase the sensitivity of MRI criteria for diagnosing multiple sclerosis without compromising specificity (2, 14). However, the presence of oligoclonal bands in cerebrospinal fluid can also be used to substitute for DIT, which has been supported by the observation that oligoclonal bands are an independent risk factor for further clinical episodes in patients with clinically isolated syndrome (3, 9, 15). Thus, cerebrospinal fluid diagnostics with the detection of oligoclonal bands is essential for patients who experienced a clinically isolated syndrome, allowing the diagnosis of multiple sclerosis when MRI scan meets criteria for DIS (2). The impact of the new McDonald criteria for the diagnosis of multiple sclerosis was in the focus of several investigations during the past year. The objective of this review is to review and to summarize these data from different cohorts and to verify if the diagnosis of multiple sclerosis has been improved using the McDonald criteria of 2017.

## Oligoclonal Bands in Multiple Sclerosis

The significance of cerebrospinal fluid examination for multiple sclerosis diagnosis decreased successively in each revision of the McDonald criteria until 2010 but still remained an important diagnostic test (2). The qualitative demonstration of two or more cerebrospinal fluid specific oligoclonal bands is the most sensitive method to show an intrathecal IgG antibody synthesis (16–18). The highest sensitivity and specificity of oligoclonal band testing can be achieved with the method of isoelectric focusing (16–18). To confirm that oligoclonal bands are exclusive to cerebrospinal fluid, paired cerebrospinal fluid and serum samples have to be analyzed in parallel and equal amounts of IgG have to be applied (2). Visualization of oligoclonal bands is preferentially performed by IgG specific antibody staining or by a general protein staining (16). Five isoelectric focusing patterns of oligoclonal bands are differentiated following the recommendations of the first European consensus on cerebrospinal fluid analysis in multiple sclerosis (16). Isoelectric focusing pattern type 1 are defined as absence of oligoclonal bands in the cerebrospinal fluid. Type 2 represents oligoclonal bands restricted to the cerebrospinal fluid (local synthesis). Type 3 means oligoclonal bands restricted to the cerebrospinal fluid and additional identical oligoclonal bands in cerebrospinal fluid and serum (local and systemic synthesis). Type 4 represents identical oligoclonal bands in cerebrospinal fluid and serum (systemic synthesis, no local synthesis). Type 5 demonstrates monoclonal bands in cerebrospinal fluid and serum (paraproteinemia, no local synthesis).

In recent studies, about 70% of patients with clinically isolated syndrome and more than 90% of patients with multiple sclerosis were tested oligoclonal bands positive (19–27). It has been demonstrated that the presence of oligoclonal bands has a positive predictive value of 97%, a negative predictive value of 84%, a sensitivity of 91%, and a specificity of 94% for developing relapsing-remitting multiple sclerosis after clinically isolated syndrome (28). Oligoclonal bands can also serve as biomarker to predict conversion from clinically isolated syndrome to multiple sclerosis (29). Studies applying older McDonald criteria to diagnose multiple sclerosis showed that the presence of oligoclonal bands in clinically isolated syndrome patients doubled the risk to develop multiple sclerosis independent of the MRI findings (3). Furthermore, two recent studies demonstrated that clinically isolated syndrome patients with oligoclonal bands were twice as likely to convert to multiple sclerosis according to McDonald criteria of 2010 as oligoclonal bands negative patients (29, 30). The probability to develop multiple sclerosis was even more pronounced when referred to quantitative intrathecal IgG synthesis (Reiber graphs) (29). On the other side, it should be noted that the finding of oligoclonal bands by isoelectric focusing is not specific for multiple sclerosis. The differential diagnosis for the presence of oligoclonal bands comprises various other autoimmune diseases such as autoimmune encephalitis, cerebral vasculitis, and neurosarcoidosis and numerous infectious diseases such as viral encephalitis, neuroborreliosis, and neurosyphilis (31–37). The exclusion of alternative diagnoses is fundamental in patients with a clinically isolated syndrome and in general in patients with suspected CNS inflammatory disease (19, 20).

## McDonald Criteria

### McDonald Criteria 2001

In 1983, the Poser criteria originally incorporated oligoclonal bands into multiple sclerosis diagnostic criteria to stress paraclinical evidence of inflammatory damage in the central nervous system (11, 38). The subsequent McDonald criteria of 2001 replaced the Poser criteria and established the use of MRI as a central tool in the diagnosis of multiple sclerosis (4, 38). The 2001 McDonald criteria demanded evidence of dissemination of lesions in both space and time which could be demonstrated clinically or by MRI supported by other paraclinical diagnostic methods like cerebrospinal fluid examination to enable the diagnosis of multiple sclerosis in patients with different clinical presentations (4, 38). DIS was defined according to the Barkhof–Tintoré MRI criteria for brain abnormalities in multiple sclerosis (three of the four: ≥1 Gd-enhancing or ≥9 T2-hyperintense lesions, ≥1 infratentorial, ≥1 juxtacortical, and ≥3 periventricular lesions), or the presence of 2 silent T2-weighted brain lesions and oligoclonal bands (4, 38–40). DIT could be demonstrated by evidence of a new contrast enhanced lesion 3 months or by a new T2-hyperintensive lesion 6 months after the initial clinical event (4, 38–40). According to the McDonald criteria of 2001 multiple sclerosis could be diagnosed in an earlier stage in patients with clinically isolated syndrome and showed high specificity (83%), sensitivity (83%), positive predictive value (75%), negative predictive value (89%), and accuracy (83%) for the risk to develop multiple sclerosis after clinically isolated syndrome (38, 41).

### McDonald Criteria 2005

In the 2005 revisions of the McDonald criteria of 2001, DIT was evident when a MRI scan, which was performed at least 30 days (instead of 90 days in the 2001 criteria) after the initial clinical event showed a new T2 lesion or a new contrast enhanced lesion was found 3 month after (6, 38). Changes of DIS criteria affected MRI spinal cord lesions, which were considered equivalent to a infratentorial brain lesion or counted as one brain lesion to reach the required number of T2 lesions (6, 38). Furthermore, enhancing spinal cord and brain lesions were equated (6, 38).

The detection of oligoclonal bands remained an additional parameter to demonstrate DIS together with at least 2 multiple sclerosis typical MRI lesions in relapsing-remitting multiple sclerosis (6, 38). The revision resulted in higher sensitivity (77%) and accuracy (86%) in the diagnosis of multiple sclerosis after clinically isolated syndrome with maintaining the high specificity (90%) of the original McDonald criteria (38, 42).

### McDonald Criteria 2010

The major achievement of the 2010 revision of the McDonald criteria was that multiple sclerosis can already be diagnosed with a single baseline MRI at the time of first clinical manifestation (5, 38, 43). DIT could be demonstrated when on MRI scan at any time asymptomatic gadolinium enhancing and non-enhancing lesions were simultaneously present or when any T2 or gadolinium enhancing lesion(s) could be found on follow up scan any time after the baseline scan (5, 38, 43). Furthermore, DIS was easier to achieve by demonstration of at least one T2 lesion in at least two of four central nervous system locations: juxtacortical, periventricular, infratentorial, and spinal cord (5, 38, 43). However, symptomatic brainstem and spinal cord lesions could neither be used for DIT nor DIS (5, 38, 43). On the other side, cerebrospinal fluid diagnostic including presentation of oligoclonal bands lost its role in supporting the diagnosis of relapsing-remitting multiple sclerosis (5, 38, 43).

The revision of the McDonald criteria of 2010 was intended to make diagnostic work-up easier and more efficient by reducing the number of MRI scans but contained pitfalls for neuroradiologist who had to differentiate between symptomatic and asymptomatic lesions (5, 38). It has also led to an earlier definite diagnosis of multiple sclerosis (25, 38). Nevertheless, clinicians were cautioned not to overemphasize MRI findings without making a thorough clinical evaluation and careful differential diagnosis, as the MRI criteria do not distinguish between multiple sclerosis and other disorders that can cause similar changes in the central nervous system (44, 45). This concern has been demonstrated in a recent study which included 168 patients with headache who presented with T2 white matter hyperintensities on brain MRI. In 2.4% of these patients MRI lesions were in contact with cortical and ventricular surfaces thus fulfilling the Barkhof-Tintoré criteria for multiple sclerosis and in 7.1% of these patients MRI lesions were at least close with having an edge within 3 mm of the surfaces (38, 46). When applying the McDonald criteria of 2010, numbers even increased to 24.4% of patients who met the imaging criteria for multiple sclerosis and 34.5% of patients whose lesions were close to cortical and ventricular surfaces (38, 46).

### McDonald Criteria 2017

One of the most important changes in the 2017 revised McDonald criteria is that oligoclonal bands can be taken as a substitute for DIT, and thus, can be used to establish the diagnosis of multiple sclerosis after the first clinical event and a single brain MRI (2, 45). These new implications based on observations demonstrating that in patients who fulfilled the criteria of DIS, the additional presence of oligoclonal bands increased the specificity and has a high positive predictive value for diagnosis of multiple sclerosis (2, 25, 30, 45, 47). Cerebrospinal fluid analysis is not only important to determine oligoclonal bands but also to exclude differential diagnosis by assessing atypical parameters such as an elevated protein concentration, pleocytosis with >50 cells/μl, or the presence of neutrophils, eosinophils, atypical cells (18, 25). Furthermore, the distinction from neuromyelitis optica spectrum disease, a demyelinating disease with overlapping clinical, imaging, and cerebrospinal fluid features, is in particular important due to the different treatment (2).

Further changes in the 2017 McDonald criteria apply to MRI activity. The 2010 McDonald criteria did not allow symptomatic brainstem or spinal cord lesions to demonstrate DIT or DIS to avoid so-called double counting. Since several studies indicated that the inclusion of symptomatic lesions increased the diagnostic sensitivity with slight affection on specificity (2, 14, 48) the 2017 McDonald criteria allow now including symptomatic and asymptomatic MRI lesions in the determination of DIS and DIT. Furthermore, in the new McDonald criteria cortical lesions are equivalent to juxtacortical lesions. Since histopathological studies have shown that cortical lesions and juxtacortical lesions are typical of multiple sclerosis and MRI techniques improved to identify these lesions, cortical lesions can now be used to fulfill MRI criteria for DIS (2, 49, 50). However, cortical lesions have to be considered carefully, since standard MRI has limited ability to detect and distinguish cortical lesions from other causes and artifacts (2).

Recently published data show that by applying the 2017 McDonald criteria, multiple sclerosis can be diagnosed more frequently at the time of first clinical event (10, 51–56). We previously investigated the diagnostic sensitivity in 325 patients with a clinical event suggestive of multiple sclerosis and found that 70 patients (22%) were diagnosed with multiple sclerosis when the 2005 criteria were applied (25). The McDonald criteria of 2010 allowed already a higher number of 136 patients (42%) to be designated as having multiple sclerosis (25). Application of the new McDonald criteria of 2017 on the same cohort allowed the diagnosis of definite multiple sclerosis in 78 additional patients (in total 214 patients; 66%) (56). Seventy-six of the 78 newly diagnosed patients with multiple sclerosis presented oligoclonal bands (56). These effects of the new McDonald criteria on earlier diagnosis of multiple sclerosis results were confirmed by additional recently published studies (summarized in Table 1).

**Table 1:** Demographic characteristics of patients diagnosed with multiple sclerosis according to the McDonald criteria of 2010 and 2017 in different studies.

**Authors**	**Country**	**Study inclusion criteria**	**Patient numbers**	**Mean age (years)**	**Females/males**	**Multiple sclerosis**		**Additional multiple sclerosis diagnoses based on new DIT criteria**	
						**McDonald 2010**	**McDonald 2017**	**Oligoclonal bands in CSF**	**Symptomatic MRI lesions**
Schwenkenbecher et al. (56)	Germany	First clinical event	325	34.2	1.8	136/325 (42%)	214/325 (66%)	76/78 (97%)	16/78 (21%)
Hyun et al. (54)	Korea	First clinical event	163	31	2.1	72/163 (44%)	124/163 (76%)	n.a. for all patients	n.a. for all patients
Habek et al. (53)	Croatia	First clinical event	113	32.1	0.4	39/113 (35%)	83/113 (74%)	43/44 (98%)	15/44 (34%)
van der Vuurst de Vries et al. (10)	Netherlands	First clinical event	229	33.5	2.7	46/180^*^ (26%)	97/180^*^ (54%)	32/51 (63%)	19/51 (37%)
Lee et al. (55)	Germany	First clinical event + DIS in MRI	290	36.4	2.1	152/290 (52%)	273/290 (94%)	121/121 (100%)	10/121 (8%)
McNicholas et al. (57)	Ireland	First clinical event + conversion to multiple sclerosis^**^ during follow up	250	33	2.6	40/250 (16%)	110/250 (44%)	61/71 (86%)	10/71 (14%)
Gobbin et al. (58)	Italy	First clinical event with follow up investigations^***^	55	30^****^	1.5	49/55 (89%)	54/55 (98%)	4/5 (80%)	1/5 (20%)
Gaetani et al. (52)	Italy	Clinically isolated syndrome^*****^ + DIS in MRI	137	31.4	2.9	0/137	113/137 (82%)	105/137 (77%)	58/137 (42%)

*Whether additional multiple sclerosis diagnoses according to McDonald 2017 based on new DIT criteria by symptomatic MRI lesions or oligoclonal bands is enlisted*.

* *MRI data available for 180/229 patients*.

** *McDonald 2010*.

*** *The McDonald criteria were compared in patients with follow up investigations*.

**** *Mean age was not described, Median age was 30 years*.

***** *Multiple sclerosis patients diagnosed according to McDonald 2010 were not included*.

Habek and colleagues investigated 113 patients with clinically isolated syndrome and found that 83 patients (74%) could be diagnosed with multiple sclerosis by applying the McDonald criteria of 2017, whereas the McDonald criteria of 2010 allowed only 39 patients (35%) to be designated as having multiple sclerosis (53). The sensitivity was higher for the new McDonald criteria (85%) as compared to the McDonald criteria of 2010 (41%), however, the specificity dropped from 85% in the criteria of 2010 to 63% with the criteria of 2017 (53). In the study of van der Vuurst de Vries and colleagues 97 of 180 patients (54%) fulfilled the McDonald criteria of 2017 in contrast to only 46 of 180 patients (26%) by applying the McDonald criteria of 2010 (10). The sensitivity was higher for the 2017 criteria than for the 2010 criteria (68 vs. 36%), but the specificity was lower (61 vs. 85%) (10). Hyun and colleagues found similar results when applying the McDonald criteria of 2017 in Korean clinically isolated syndrome patients and described higher sensitivity (88.8%) and accuracy (70.6%) but lower specificity (43.1%) compared with the 2010 McDonald criteria (sensitivity: 53.1%, accuracy 69.2%, specificity 59.5%) for prediction of conversion (54). Again, more clinically isolated syndrome patients could be diagnosed with multiple sclerosis by using the new criteria (76%) as compared to 2010 criteria (44%) (54). Lee and colleagues investigated a large cohort of 290 clinically isolated syndrome patients and identified 52% of patients with the diagnosis of multiple sclerosis according to the McDonald criteria of 2010 (55). The application of the McDonald criteria of 2017 increased the number of multiple sclerosis patients to 94%, thus leaving only 6% of patients with the diagnosis of clinically isolated syndrome (55). The high number of multiple sclerosis patients differs from the other described cohorts and might be explained by the fact that Lee and colleagues included only patients with clinically isolated syndrome who fulfilled MRI criteria for DIS in their cohort (55). In contrast, in the cohort of van der Vuurst de Vries and colleagues only 54% of clinically isolated syndrome patients fulfilled DIS criteria (10). Similar results to Lee and colleagues were found by Gaetani and colleagues who investigated clinically isolated syndrome patients only (excluding patients diagnosed with multiple sclerosis according to the McDonald criteria of 2010) in combination with fulfilled DIS (52). Eighty-two percent of these clinically isolated syndrome patients could be diagnosed with multiple sclerosis by applying the McDonald criteria of 2017 (52). McNicholas and colleagues compared the time to diagnosis when applying the 2017 McDonald criteria in a cohort of patients who had been diagnosed with multiple sclerosis according to the McDonald criteria of 2010 and found a significant improvement (57). The median time to diagnosis could be reduced from 7.4 months (McDonald 2010) to 2.3 months (McDonald 2017) (57). In total, the 2017 McDonald criteria allowed 142/250 patients (57%) to receive an earlier diagnosis. CSF data were available in 200/250 patients and the authors found that the presence of oligoclonal bands allowed an earlier diagnosis in 127/200 patients. The authors describe that 40 of 250 patients (16%) initially fulfilled the 2010 McDonald criteria for multiple sclerosis in an outpatient setting. The 2017 McDonald criteria would have allowed 110 patients (44%) to be diagnosed with multiple sclerosis. However, the authors describe that the extent of investigations carried out prior to first review in this group varied greatly according to the referral source. Gobbin and colleagues investigated a cohort of 55 patients with a first demyelinating event. Forty-nine of these 55 patients fulfilled the multiple sclerosis diagnostic criteria according to McDonald 2010 after a follow-up of 7 months (0–73) (58). A higher number of 54 of these patients were diagnosed with multiple sclerosis after 1 month (0–64) when the 2017 McDonald criteria were applied.

Five studies calculated the sensitivity for conversion to multiple sclerosis by applying the 2017 McDonald criteria which ranged from 68 to 100% (10, 53–55, 58). The specificity was low in these studies and ranged from 13.8 to 63% for the new criteria. However, a low specificity is expected when retrospectively testing newer and more inclusive criteria compared to older and less inclusive diagnostic criteria. Furthermore, there are several bias due to limitations that need to be critically discussed: the studies included a low number of patients and short time of follow-up; the exposure to disease-modifying drugs during follow-up needs to be considered since it could lead to a delay in the second clinical manifestation or in the appearance of new MRI T2 or contrast enhancing lesions. These limitations within a short time period might be a reason for a reduced disease activity limiting the frequency of conversion rates to definitive multiple sclerosis. Thus, future prospective studies with larger cohorts are needed to evaluate the specificity of the new McDonald criteria.

### Pitfalls by Applying McDonald Criteria 2017

While the McDonald criteria were developed to establish a consensus for multiple sclerosis diagnosis, limitations are related to alternative inflammatory central nervous system disorders (2, 45). It is challenging to diagnose multiple sclerosis in patients who indeed fulfill the diagnostic criteria but present with uncommon clinical syndromes. Since the McDonald criteria are primarily to be applied in patients with a typical clinically isolated syndrome, cases of an atypical clinical presentation are challenging and require expertise in multiple sclerosis in order to make a reliable diagnosis or an alternative diagnosis (2, 45). MRI findings and clinical presentations can be misleading in patients with migraine and vascular risk comorbidities (45). The specificity for multiple sclerosis diagnosis may also be improved by considering the perivascular distribution pattern of multiple sclerosis lesions including the so called “central vein sign” to differentiate vascular pathology and other inflammatory CNS lesions from multiple sclerosis, the identification of callosal lesions, thorough assessment of spinal cord lesions and at least two different MRI sequences to confirm lesions (39, 45, 59). Furthermore, the detection of oligoclonal bands can support the diagnosis of multiple sclerosis or when absent should lead to a thorough re-evaluation (45). On the other hand, it could also be shown that initially oligoclonal bands negative multiple sclerosis patients were eventually tested positive in a follow-up spinal tap (60, 61). Therefore, a second lumbar puncture is desirable in patients with a questionable multiple sclerosis diagnosis and in patients with clinically isolated syndrome at high risk to develop multiple sclerosis (25). However, although highly prevalent, oligoclonal bands are not specific for multiple sclerosis and can be also detected in numerous other autoimmune and infectious central nervous system diseases (31–37).

## Conclusion

**Figure 1:**
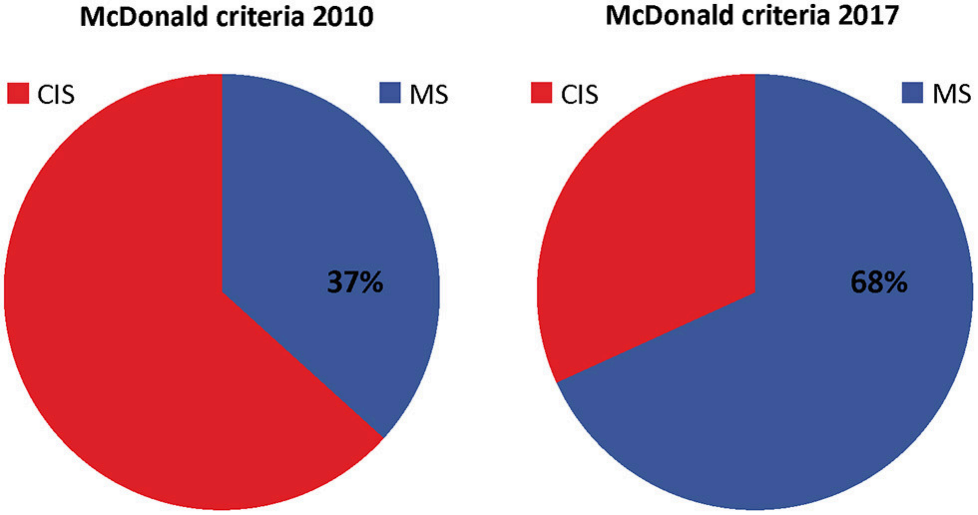
Distribution of patients diagnosed with clinically isolated syndrome (CIS) and multiple sclerosis (MS) according to McDonald criteria of 2010 and 2017. Distribution was created with pooled data from Schwenkenbecher et al. (56); Hyun et al. (54), Habek et al. (53), van der Vuurst de Vries et al. (10), Lee et al. (55), and McNicholas et al. (57).

The 2017 McDonald criteria were developed to allow a more rapid diagnosis of multiple sclerosis and achieved their goal at an impressive extent (Figure 1). The main effect was due to the implementation of oligoclonal bands as a substitute for dissemination in time. Alternative less technically demanding and cost saving biomarker to oligoclonal bands might play a role in a future revision of the McDonald criteria. However, limitations of the 2017 McDonald criteria when applied on atypical clinical manifestations and misleading MRI findings should be carefully considered.

## Data Availability

All datasets generated for this study are included in the manuscript and/or the supplementary files.

## Author Contributions

PS, UW, FK, SG, K-WS, MW, MS, and TS provided expertise for the conception and design of the study, contributed to the drafting and approved the final version of this manuscript.

### Conflict of Interest Statement

The authors declare that the research was conducted in the absence of any commercial or financial relationships that could be construed as a potential conflict of interest.
